# Higher Frequency of Hospital-Acquired Infections but Similar In-Hospital Mortality Among Admissions With Alcoholic Hepatitis at Academic vs. Non-academic Centers

**DOI:** 10.3389/fphys.2020.594138

**Published:** 2020-12-03

**Authors:** Muhammad Waleed, Mohamed A. Abdallah, Yong-Fang Kuo, Juan P. Arab, Robert Wong, Ashwani K. Singal

**Affiliations:** ^1^Department of Medicine, University of South Dakota Sanford School of Medicine, Sioux Falls, SD, United States; ^2^Department of Biostatistics, University of Texas Medical Branch at Galveston, Galveston, TX, United States; ^3^Departamento de Gastroenterología, Escuela de Medicina, Pontificia Universidad Católica de Chile, Santiago, Chile; ^4^Division of Gastroenterology and Hepatology, Alameda Health System Highland Hospital, Oakland, CA, United States; ^5^Division of Transplant Hepatology, Avera Transplant Institute, Sioux Falls, SD, United States

**Keywords:** academic, outcomes, acute on chronic liver failure (ACLF), cirrhosis, organ failure

## Abstract

**Background:**

Alcoholic hepatitis (AH) is a unique syndrome characterized by high short-term mortality. The impact of the academic status of a hospital (urban and teaching) on outcomes in AH is unknown.

**Methods:**

National Inpatient Sample dataset (2006–2014) on AH admissions stratified to academic center (AC) or non-academic center (NAC) and analyzed for in-hospital mortality (IHM), hospital resource use, length of stay in days (d), and total charges (TC) in United States dollars (USD). Admission year was stratified to 2006–2008 (TMI), 2009–2011 (TM2), and 2012–2014 (TM3).

**Results:**

Of 62,136 AH admissions, the proportion at AC increased from 46% in TM1 to 57% in TM3, Armitage trend, *p* < 0.001. On logistic regression, TM3, younger age, black race, Medicaid and private insurance, and development of acute on chronic liver failure (ACLF) were associated with admission to an AC. Of 53,264 admissions propensity score matched for demographics, pay status, and disease severity, admissions to AC vs. NAC (26,622 each) were more likely to have liver disease complications (esophageal varices, ascites, and hepatic encephalopathy) and hospital-acquired infections (HAI), especially *Clostridioides difficile* and ventilator-associated pneumonia. Admissions to AC were more likely transfers from outside hospital (1.6% vs. 1.3%) and seen by palliative care (4.8% vs. 3.3%), *p* < 0.001. Use of endoscopy, dialysis, and mechanical ventilation were similar. With similar IHM comparing AC vs. NAC (7.7% vs. 7.8%, *p* = 0.93), average LOS and number of procedures were higher at AC (7.7 vs. 7.1 d and 2.3 vs. 1.9, respectively, *p* < 0.001) without difference on total charges ($52,821 vs. $52,067 USD, *p* = 0.28). On multivariable logistic regression model after controlling for demographics, ACLF grade, and calendar year, IHM was similar irrespective of academic status of the hospital, HR (95% CI): 1.01 (0.93–1.08, *p* = 0.70). IHM decreased over time, with ACLF as strongest predictor. A total of 63 and 22% were discharged to home and skilled nursing facility, respectively, without differences on academic status of the hospital.

**Conclusion:**

Admissions with AH to AC compared to NAC have higher frequency of liver disease complications and HAI, with longer duration of hospitalization. Prospective studies are needed to reduce HAI among hospitalized patients with AH.

## Introduction

Alcohol contributed to 48% of cirrhosis-related deaths in the United States in 2017, and alcohol-associated liver disease (ALD) accounts for 27% of these (GBD 2017 Cirrhosis Collaborators, 2020). Alcohol-associated liver disease presents as a spectrum of injuries to the liver, including isolated fatty liver disease, alcoholic hepatitis (AH), and cirrhosis (Chacko and Reinus, 2016).

With the recent advances in the treatment of hepatitis B and C, ALD has emerged as the leading indication for liver transplantation (LT; Williams, 2008; Cholankeril and Ahmed, 2018). There are also increasing trends on alcohol consumption especially drinking in younger individuals (Siegel et al., 2011a,b). Of approximately $250 billion spent by the United States annually on alcohol-related problems, a significant proportion is spent on taking care of patients with AH and alcohol-associated cirrhosis (Singal et al., 2020). Additionally, AH most often occurs in individuals aged 40–60 years, with the majority of these individuals contributing to the most productive contingent of the workforce (Thompson et al., 2018). Taken together, these trends in alcohol consumption contribute toward burden on social, economic, and health care systems (Jinjuvadia and Liangpunsakul, 2015).

AH is a distinct clinical entity among ALD patients and presents with jaundice and acute on chronic liver failure (ACLF; Altamirano et al., 2017). With limited therapeutic options for AH, it has a potential for high short-term mortality of 15–40% within 28 days of presentation (Altamirano et al., 2017). Given the very high prevalence of AH among hospitalized patients with decompensated ALD, patients with AH are treated by non-hepatology providers such as hospitalists, internists, and gastroenterologists at both academic (AC) and non-academic centers (NAC; Singal and Anand, 2013). Data on outcomes of AH patients comparing hospitalizations at AC with those hospitalized at NAC are scanty. In this study, we aim to compare characteristics and outcomes of AH patients hospitalized to AC vs. NAC using the National Inpatient Sample (NIS) Database.

## Patients and Methods

### Study Design and Data Source

The Healthcare Cost and Utilization Project NIS database on hospitalizations between 2006 and 2014 in the US was used for this study. The largest inpatient database in the US representing 20% stratified sample of all hospital discharges from 46 states (approximately 97% of the United States population), NIS database is developed and maintained by the Agency for Healthcare Research and Quality. It contains data from over 7 million hospital discharges annually, yielding national estimates of hospital inpatient stays. The NIS includes up to 25 International Classification of Diseases (ICD) discharge diagnoses or procedures. The database also provides information on patient demographics, payer status, comorbidities, in-hospital mortality (IHM), hospital characteristics (region, urban, or rural location, and teaching status), length of stay (LOS) in days, and total hospital charges in the United States dollars (USD). Given that the NIS is completely de-identified and publicly available, institutional review board (IRB) approval was not required for this study.

### Study Population

Our study population included hospitalizations with discharge diagnosis of AH identified using the ICD-09 diagnosis code 571.1 for AH (Supplementary Table 1). The study population was stratified to three time frames: 2006–2008 (TM1), 2009–2011 (TM2), and 2012–2014 (TM3).

### Definitions

#### Academic vs. Non-Academic Centers

Hospitals in urban locations with teaching status were classified as AC and the remaining as NAC.

#### Acute on Chronic Liver Failure

Acute on chronic liver failure was defined per North American Consortium for the Study of End-Stage Liver Disease (NACSELD) Criteria, as the presence of two or more extrahepatic organ failures [cardiovascular (CV), respiratory, renal, and brain] in patients with cirrhosis (Bajaj et al., 2014; Allen et al., 2016; O’Leary et al., 2018; O’Leary et al., 2019). Organ failures were defined using the ICD-09 codes (Supplementary Table 1; Allen et al., 2016; Singal et al., 2020). Severity of ACLF was graded to ACLF-1, ACLF-2, and ACLF-3 in the presence of two, three, or four extrahepatic organ failures.

#### Organ Failures

International Classification of Diseases-09 procedure codes were used to define CV failure by (need for central venous pressure, pulmonary artery/wedge pressure, use of arterial line, the presence of septic shock, or severe sepsis); respiratory failure (using code for mechanical ventilation); renal failure (need for hemodialysis or diagnosis code of acute renal failure), and brain failure (diagnosis code for hepatic encephalopathy) (Supplementary Table 1).

#### Liver Disease Complications

Ascites, variceal bleeding, hepatic encephalopathy, community-acquired infections [CAI; urinary tract infection, spontaneous bacterial peritonitis (SBP), pneumonia, skin, and soft tissue infections], or hospital-acquired infections (HAI; *Clostridioides difficile*, ventilator-associated pneumonia, central line-associated bloodstream infection, catheter-associated urinary tract infection) were identified using discharge ICD-09 diagnoses codes (Supplementary Tables 1, 2).

### Study Outcomes

Primary outcomes of the study were IHM, LOS, and total hospital charges. Secondary outcomes were (a) use of hospital resources (blood transfusion, hemodialysis, endoscopic intervention, ventilator support, liver transplant, and palliative care) as identified using the ICD-09 procedure codes and (b) discharge destination of survivors (home with or without home health care, short-term hospitals, and intermediate/long-term nursing care facility) as available from the dataset.

### Statistical Analyses

Baseline characteristics were compared for AC vs. NAC using chi-square and Student *t* test for categorical and continuous variables, respectively. Hospitalization trends over period were analyzed using Cochran-Armitage trend test. Hospitalizations to AC or NAC were propensity score matched (1:1) on variables associated with admission to AC, as identified by logistic regression model. Baseline characteristics were also compared in the matched cohort. Admissions to AC were compared to NAC on primary and secondary outcomes. Logistic regression analysis model was built to determine predictors of IHM and reported as odds ratio (OR) with 95% confidence interval (CI). *p* values < 0.05 were considered significant. SAS version 9.4 (SAS Institute, Cary, NC) was used for statistical analyses.

## Results

### Study Population

**FIGURE 1:**
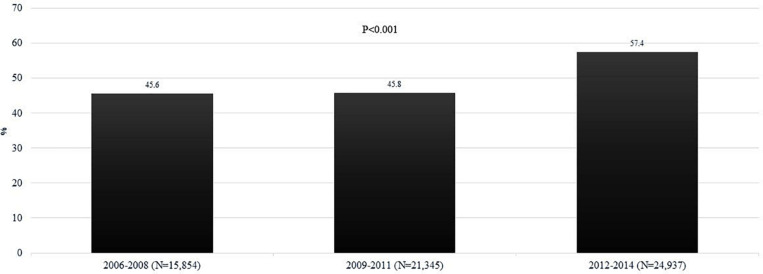
Trends on the proportion of patients admitted with AH among admission to AC for the time periods 2006–2008 (TM1), 2009–2011 (TM2), and 2012–2014 (TM3). AC, academic centers; NAC, non-academic centers; AH, alcoholic hepatitis.

A total of 62,136 admissions with discharge diagnosis of AH were admitted during 2006–2014, with 31,321 (50.5%) admitted to an AC. Over the period, frequency of admissions to AC increased from 45.6% in TM1 to 45.8% in TM2 to 57.4% in TM3, *p* < 0.001 (Figure 1). The proportion of patients with ACLF over time for AH admissions was higher for TM1 but similar for TM2 and TM3 comparing AC vs. NAC: 6.8% vs. 5.8% (*p* < 0.001); 11.2% vs. 11.9% (*p* = 0.79); and 10.8% vs. 10.9% (*p* = 0.79), respectively.

#### Baseline Characteristics Admissions With AH: AC vs. NAC

Admissions to AC compared to NAC differed on demographics (younger age and more likely to be females and minorities in AC). Further, admissions to AC were less likely to be electively admitted and associated with Medicare pay source. About 72% admissions were associated with underlying alcohol-associated cirrhosis, without difference between admissions to AC or NAC (Table 1). Admissions to AC were associated with more severe disease with ACLF (12% vs. 9%, *p* < 0.001) and various organ failures (Table 1). On logistic regression analysis, time period, younger age, African American race, pay source other than Medicare, and the presence of ACLF were associated with admission to an AC (Table 2).

**TABLE 1:** Comparison of baseline characteristics of admissions with diagnosis of alcoholic hepatitis (AH) to academic centers (AC) vs. non-academic centers (NAC).

Variables	Unmatched AH cohort			Matched AH cohort		
	AC (*N* = 31,321)	NAC (*N* = 30,815)	*p* value	AC (*N* = 26,622)	NAC (*N* = 26,622)	*p* value
Age in years (mean ± SD)	49 ± 10	50 ± 10	<0.001	49.7 ± 10.2	49.8 ± 10.2	0.34
Females (%)	34	33	<0.029	33	33	0.3
CA: AA: HI: OT (%)	67: 12: 15: 6	77: 7: 11: 5	<0.001	64:7: 12: 17	65: 7: 11: 17	0.2
Comorbidities, (mean ± SD)	7 ± 3.2	7.4 ± 3.3	<0.001	7.3 ± 3.3	7.3 ± 3.2	0.07
Obesity (%)	6.4	6.3	0.61	6.1	6.6	< 0.02
Alcoholic cirrhosis (%)	72	72	0.89	72	72	0.41
Elective admissions (%)	4.5	5.7	<0.001	4.9	4.6	0.12
Pay source (MC: MD: Self: Pvt. (%)	16: 30: 28: 26	20: 24: 29: 27	<0.001	17:27: 28: 28	18: 27: 29: 27	0.7
ACLF (%)	12.1	9.3	<0.001	9.9	10	0.64
Pulmonary failure (%)	10.1	8	<0.001	8.4	8.4	0.75
Cardiovascular failure (%)	6.2	4.5	<0.001	4.7	4.9	0.28
Brain failure (%)	29.2	29.3	0.81	29.6	28.3	< 0.002
Renal failure (%)	9.9	7.6	<0.001	8.2	8.2	0.79

*CA, Caucasian; AA, African American; HI, Hispanic; OT, Other; MC, Medicare; MD, Medicaid; Pvt, private; ACLF, acute on chronic liver failure; HR, hazard ratio; CI, confidence interval; OR, odds ratio; NAC, non-academic center.*

**TABLE 2:** Factors associated with admission of patients with alcoholic hepatitis to academic centers.

Variable	Odds ratio	95% CI	*p* value
Age	0.99	0.989–0.993	<0.001
2009–2011 vs. 2006–2008	0.99	0.93–1.06	0.14
2012–2014 vs. 2006–2008	1.61	1.52–1.72	<0.001
AAvs. C	2.01	1.88–2.15	<0.001
H vs. C	1.40	1.32–1.48	0.76
Female vs. male	1.02	0.98–1.06	0.27
Medicaid vs. Medicare	1.31	1.24–1.40	<0.001
Pvt. insurance vs. Medicare	1.15	1.09–1.22	<0.001
ACLF	1.27	1.20–1.35	<0.001

*AA, African American; C, Caucasian; H, Hispanic; ACLF, acute on chronic liver failure; CI, confidence interval.*

#### Matching for Admissions to AC or NAC

##### Baseline characteristics

Using propensity score analysis, admissions to AC were matched (1:1) with admissions to NAC for age, race, pay source, time period of admission, and the presence of ACLF. Matched cohort included 53,264 admissions (26,622 each to AC or NAC). Baseline characteristics comparing admissions to AC vs. NAC in this matched cohort were similar except for obesity (6.1% vs. 6.6%, *p* < 0.02), Table 1. Admissions to AC were more often admitted as a transfer from another hospital (1.6% vs. 1.1%), *p* < 0.001.

##### Complications and organ failure

**FIGURE 2:**
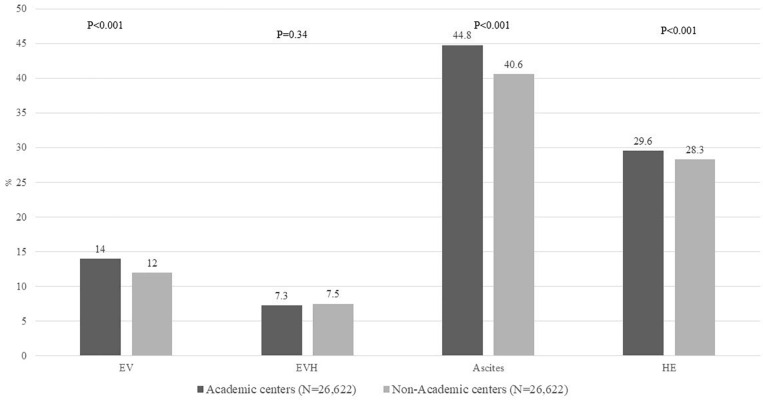
Proportion of all admissions with AH on liver disease complications including EV, EVH, ascites, and HE, comparing admissions to AC vs. NAC. AH, alcoholic hepatitis; EV, esophageal varices; EVH, esophageal variceal hemorrhage; HE, hepatic encephalopathy; AC, academic centers; NAC, non-academic centers.

About 10% admissions were associated with ACLF at or during admission with no differences comparing admissions to AC or NAC (9.9% vs. 10%, *p* = 0.64). Among 5292 AH patients with ACLF (2630 at AC), distribution to ACLF grades to grades 1, 2, and 3 comparing NAC vs. AC were 70.5% vs. 71.1%, 24.3% vs. 23.3%, and 5.2% vs. 5.6%, respectively, *p* = 0.89. Similarly, all the organ failures were similar irrespective of academic status of the hospital, except brain failure which was more frequent among admissions to AC (30% vs. 28%, *p* < 0.001), Table 1. Of liver disease complications, admissions to AC were more often associated with liver disease complications on ascites (45% vs. 41%), hepatic encephalopathy (30% vs. 28%), and esophageal varices (14% vs. 12%), *p* < 0.001 for all analyses. However, there was no difference on variceal bleeding (7.3% vs. 7.5%, *p* = 0.34), Figure 2.

##### Infections

**FIGURE 3:**
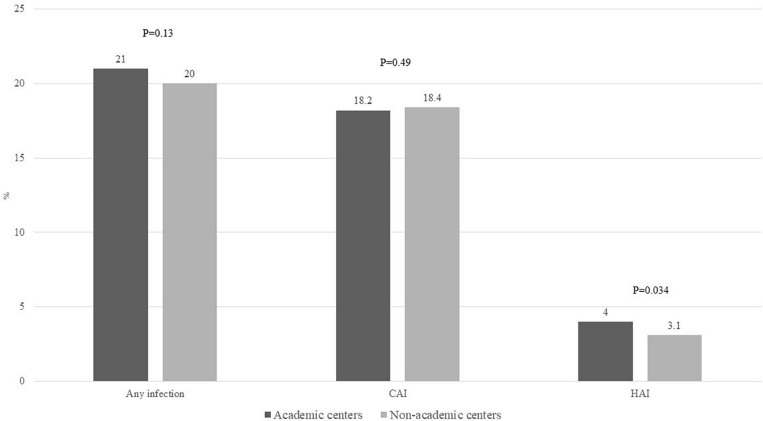
Proportion of admissions with alcoholic hepatitis to academic or non-academic centers complicated by any infection, CAI, and HAI. CAI, community-acquired infections; HAI, hospital-acquired infections.

About 20% of admissions were associated with infection, without difference comparing admissions to AC vs. NAC (21% vs. 20%, *p* = 0.13), Figure 3. On analyzing the infection source whether acquired in the community or hospital, admissions to AC were more likely to develop HAI (4% vs. 3.1%, *p* = 0.034). However, there was no difference in CAI (18.2% vs. 18.4%, *p* = 0.49), Figure 3. Based on admission as transfer from outside hospital, the frequency of CAI was similar (18.7% vs. 18.4%, *p* = 0.82). However, transfers from outside hospital were associated with higher frequency of HAI (5.6% vs. 3.6%, *p* = 0.002), Supplementary Figure 1.

Of the HAI, *C. difficile* infection and ventilator-associated pneumonia were more frequent in AC vs. NAC, 2.6% vs. 1.8% and 0.18% vs. 0.08%, respectively, *p* < 0.001 for both analyses (Supplementary Figure 2A). Of CAI, urinary tract infection was most common in about 13% patients, without differences based on academic status of admitting hospital. Similarly, SBP and pneumonia were similar comparing admissions to AC with NAC. In contrast, skin and subcutaneous infections were more common among admissions to NAC (3.8% vs. 3.4%, *p* < 0.001), Supplementary Figure 2B.

### Primary Outcomes

#### In-Hospital Mortality

**FIGURE 4:**
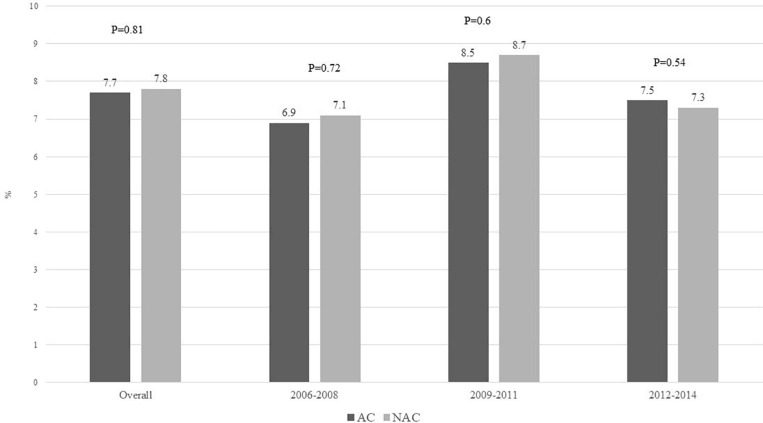
In-hospital mortality comparing admissions with alcoholic hepatitis at academic vs. non-academic centers during 2006–2008, 2009–2011, and 2012–2014.

A total of 4110 (7.7%) of admissions were associated with IHM, with no difference comparing admissions to AC vs. NAC (7.7% vs. 7.8%, *p* = 0.93) (Figure 4). In-hospital mortality comparing admissions to AC vs. NAC remained similar for TM1 (6.9% vs. 7.1%, *p* = 0.72), TM2 (8.5% vs. 8.7%, *p* = 0.6), and TM3 (7.5% vs. 7.3%, *p* = 0.54) (Figure 4). Moreover, for patients with concomitant AH and ACLF at the time of admission, IHM was found similar for AC vs. NAC (43.7% vs. 42.7%, *p* = 0.47). Patients who did not undergo LT had higher IHM than patients who underwent LT, 8.2% vs. 4.5%, *p* = 0.32. IHM was higher among admissions from other hospitals compared to admissions from within the admitting center (13.4% vs. 8.2%, *p* < 0.001), Supplementary Figure 3A.

On multivariate logistic regression analysis, the presence of ACLF had strongest association with IHM with 20 folds higher IHM in the presence of ACLF. Other predictors were older age and transfer from outside hospital (Table 3). Conversely, Hispanics were 15% less likely to die during hospitalization, and the IHM decreased by about 20% in 2012–2014 compared to admissions during 2006–2008. After controlling for all these variables, academic status of the admitting hospital was not associated with IHM, 1.00 (0.93–1.08, *p* = 0.93), Table 3.

**TABLE 3:** Predictors of in-hospital mortality among admissions with alcoholic hepatitis.

Effect	Hazard ratio	95% CI	*p* value
Age	1.03	1.026–1.035	<0.0001
2009–2011 vs. 2006–2008	0.95	0.83–1.09	0.16
2012–2014 vs. 2006–2008	0.81	0.71–0.92	<0.0001
Transfer from OSH	1.41	1.01–1.98	0.046
Females vs. males	1.00	0.92–1.09	0.25
AA vs. C	0.86	0.74–1.02	0.34
H vs. C	0.85	0.75–0.97	<0.0001
ACLF	20	18.5–21.7	<0.0001
ACvs. NAC	1.003	0.93–1.08	0.20

*AA, African American; C, Caucasian; H, Hispanic; ACLF, acute on chronic liver failure; AC, academic centers; NAC, non-academic centers; OSH, outside hospital.*

#### Length of Stay and Total Hospital Charges

**FIGURE 5:**
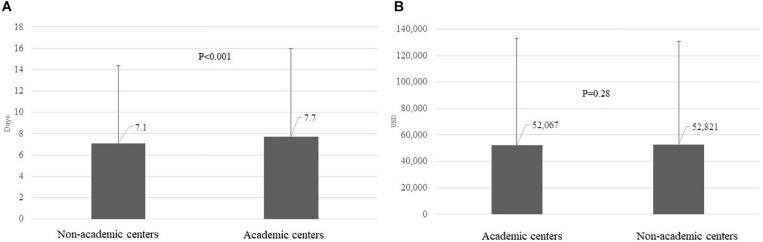
LOS (mean and S.D.) in days **(A)** and total hospital charges in USD **(B)** per admission among AH admissions, comparing admissions to AC vs. NAC. LOS, length of stay; USD, US dollars; AH, alcoholic hepatitis; AC, academic centers; NAC, non-academic centers.

Mean (SD) LOS among admissions with discharge diagnosis of AH was 7.4 (7.8) days, longer among admissions to AC compared with admissions to NAC (7.7 vs. 7.1 days, *p* < 0.001), Figure 5A. Moreover, AH admissions to AC underwent higher number of procedures than NAC admissions, 2.3 vs. 1.9 *p* < 0.001. However, there was no difference on mean total hospital charges per hospitalization comparing admissions to AC vs. NAC, $52,067 vs. $52,821, *p* = 0.28 (Figure 5B).

### Secondary Outcomes

#### Use of Hospital Resources

**FIGURE 6:**
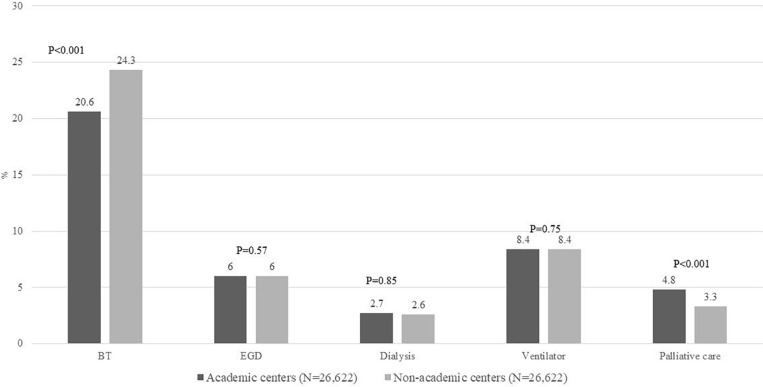
Proportion of all admissions with AH on hospital resource utilization including BT, EGD, dialysis, ventilator use, and palliative care consult comparing admissions to AC vs. NAC. BT, blood transfusions; EGD, esophagogastroduodenoscopy; AH, alcoholic hepatitis; AC, academic centers; NAC, non-academic centers.

Blood transfusion was the most frequently used hospital resource in about 22% admissions, with higher use among NAC compared to admissions at AC (24% vs. 21%, *p* < 0.001). In contrast, palliative care consultation was more often obtained among admissions to AC (4.8% vs. 3.3%, *p* < 0.001). There were no differences on use of endoscopy, hemodialysis, or mechanical ventilation (Figure 6). A total of 63 AH-related admissions were associated with receipt of liver transplantation, 61 at AC and remaining 2 at NAC, *p* < 0.001. Receipt of LT was associated with reduced IHM (4.5% vs. 8.2%); however, this difference was not significant, *p* = 0.32, given small sample size of 63 liver transplants. Admissions as transfer from another hospital compared to those admitted directly underwent similar (mean ± SD) number of procedures (2.2 ± 2.5 vs. 2.1 ± 2.5, *p* = 0.79), Supplementary Figure 3B.

#### Discharge Disposition of Survivors

**FIGURE 7:**
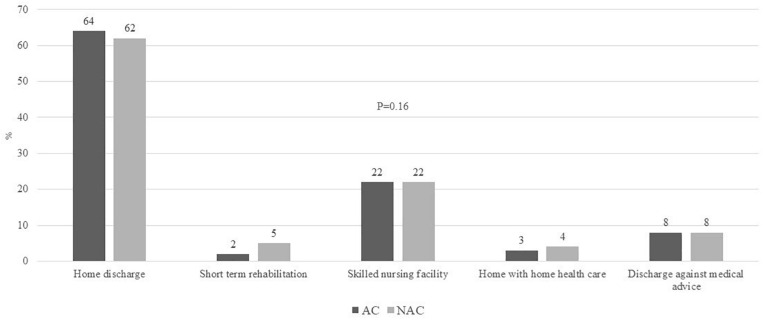
Proportion of all admissions with AH on post-discharge disposition including home discharge, short-term rehabilitation, skilled nursing facility, home with home health care, discharge against medical advice, comparing admissions to AC vs. NAC. AH, alcoholic hepatitis; AC, academic centers; NAC, non-academic centers.

Admissions with discharge diagnosis of AH who survived the hospitalization were discharged to home in 63%, short-term rehabilitation in 3.5%, skilled nursing facility in 22%, and home with home health care in 3.5%. There were no differences on discharge disposition comparing admissions to AC vs. NAC (Figure 7).

## Discussion

The main findings of our study are that AH admissions in the United States (a) are increasing to AC compared to NAC, (b) have higher prevalence of liver disease and infectious complications, and (c) have longer length of hospital stay, in spite of similar IHM in AC compared to NAC.

Higher disease severity with liver disease complications, infections, and development of ACLF are potential reasons for this finding as also observed in this study. In an earlier study using the NIS database, we have also shown increasing frequency of admissions of patients with discharge diagnosis of cirrhosis who developed ACLF at or during hospitalization (Singal et al., 2020a). Another speculated reason may be potential coding error in AC vs. NAC and underestimation of the disease severity or comorbidities (Rangachari, 2007).

In an earlier study using the NIS database, infectious and other liver disease complications have been shown to be associated with higher mortality among patients with AH (Liangpunsakul, 2011; Jinjuvadia and Liangpunsakul, 2015). Studies have also shown higher mortality among hospitalizations with liver disease and complicated by HAI as compared to admissions with CAI (Bajaj et al., 2012; Arefian et al., 2016; Singal et al., 2020a). Patients with transfer from outside hospital should have low threshold for infection diagnosis and consider nosocomial or HAI, as they have implications on how we choose presumptive antibiotic coverage pending culture results.

Although in this study AH admissions to AC compared to those at NAC had higher frequency of liver disease complications and HAI, the IHM was similar irrespective of academic status of the hospital. Although exact reasons for these findings remain to be determined, it could be speculated to be due to more frequent availability of subspecialty expertise, multidisciplinary teams, and facilities with infrastructure at AC compared to NAC (Allison et al., 2000). Moreover, similar post-discharge disposition of patients from AC vs. NAC in spite of sicker population at AC may be due to the same reasons. Previous studies have also reported better outcomes for patients admitted to AC with complex surgical diseases and medical conditions, including myocardial infarction and pneumonia (Allison et al., 2000; Hayanga et al., 2010).

Health care resource utilization and estimated hospital costs with AH are on the rise (Thompson et al., 2018). We found similar use of hospital resources (other than less blood transfusions and more palliative care consults) between AC and NAC. A possible reason for less blood transfusions in AC could be the early availability of expert intervention from gastroenterology and interventional radiology for control of gastrointestinal bleeding in these patients. Further, longer LOS among admissions to AC for other medical and surgical conditions is likely due to sicker and complex cases at AC and is similar to other reports (Khuri et al., 2001; Dimick et al., 2004; Hayanga et al., 2010; Singal et al., 2020a). Interestingly, in spite of higher number of procedures and LOS at AC compared to NAC, there was no difference on hospitalization costs. Although overall palliative care use was low in these patients as reported in other studies, (Poonja et al., 2014; O’Leary et al., 2020; Singal et al., 2020a) its more frequent use at AC may likely explain similar use of hospital resources and total costs. Further, availability of trainee residents and fellows may have potentially reduced the cost of care and at the same time provided better care to sicker and complex patients.

The national database with large sample size and propensity-matched analysis for disease severity are potential strengths of our study. However, the authors suggest a cautious approach on interpreting these results given several limitations of the study. For example, there could be coding errors in adjudicating the discharge diagnoses given this is a database study with study population identified using the ICD-09 codes. Hence, it may be possible that patients with decompensated ALD and true AH may have been included. Further, NIS database stratifies admissions without linkage to a given patient, limiting identification of previous readmissions. Unavailability of laboratory values during the hospitalization and admissions to Veteran hospital systems are some other limitations of this study.

In summary, admissions with AH in the United States are increasingly being admitted to an AC and as transfer from other hospitals. In spite of longer hospitalizations, higher frequency of liver disease complications, and HAI, admissions to AC as compared to NAC are associated with similar IHM and hospitalization-related costs. These novel findings have epidemiological implications and suggest future large prospective studies overcoming limitations of database including NIS database to validate these findings and examine its mechanisms.

## Data Availability Statement

The raw data supporting the conclusions of this article will be made available by the authors, without undue reservation.

## Ethics Statement

Ethical review and approval was not required for the study on human participants in accordance with the local legislation and institutional requirements. Written informed consent for participation was not required for this study in accordance with the national legislation and the institutional requirements.

## Author Contributions

AS conceived the study idea and designed the study. MW and MA wrote the manuscript draft. Y-FK performed the statistical analyses. RW provided important intellectual input. All the authors reviewed the final version and approved it for submission.

## Conflict of Interest

The authors declare that the research was conducted in the absence of any commercial or financial relationships that could be construed as a potential conflict of interest.
