# Supplementary figures and images for: Higher Frequency of Hospital-Acquired Infections but Similar In-Hospital Mortality Among Admissions With Alcoholic Hepatitis at Academic vs. Non-academic Centers
Source: Front Physiol. 2020 Dec 3;11:594138. doi: 10.3389/fphys.2020.594138 (PMC7744884; doi:10.3389/fphys.2020.594138)

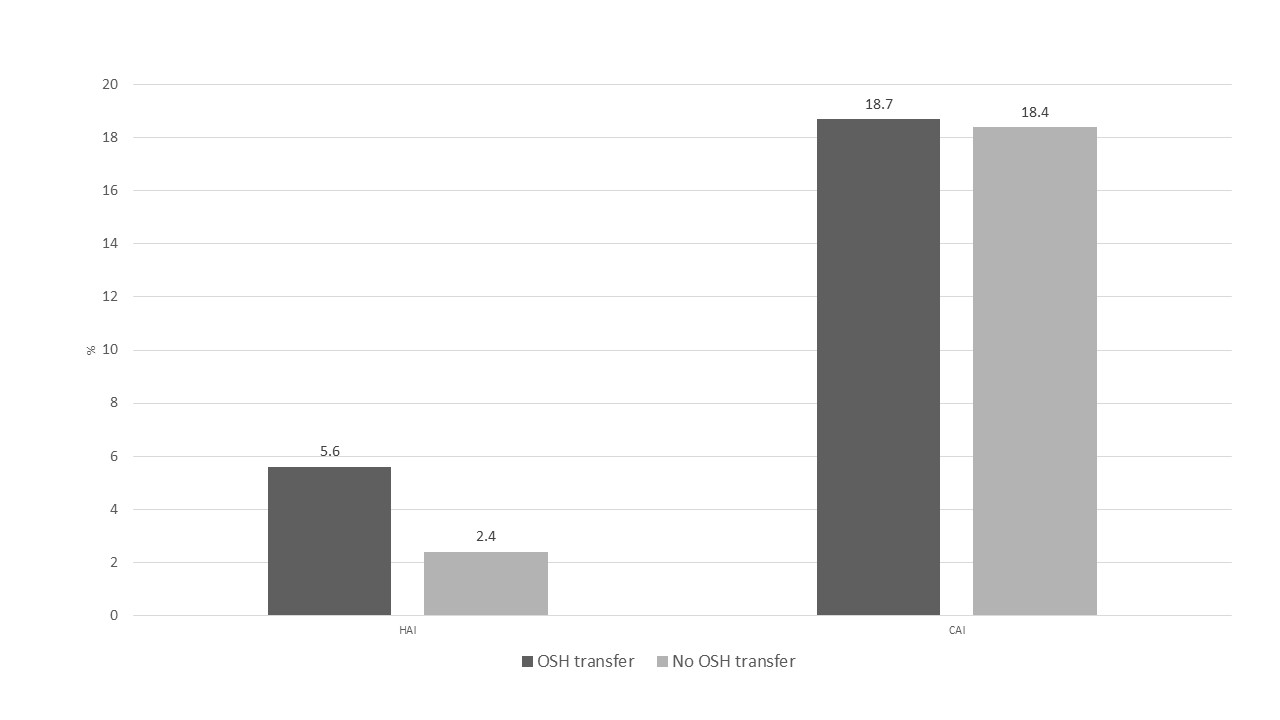

Supplement: Supplementary file 1 [file Image_1.JPEG]

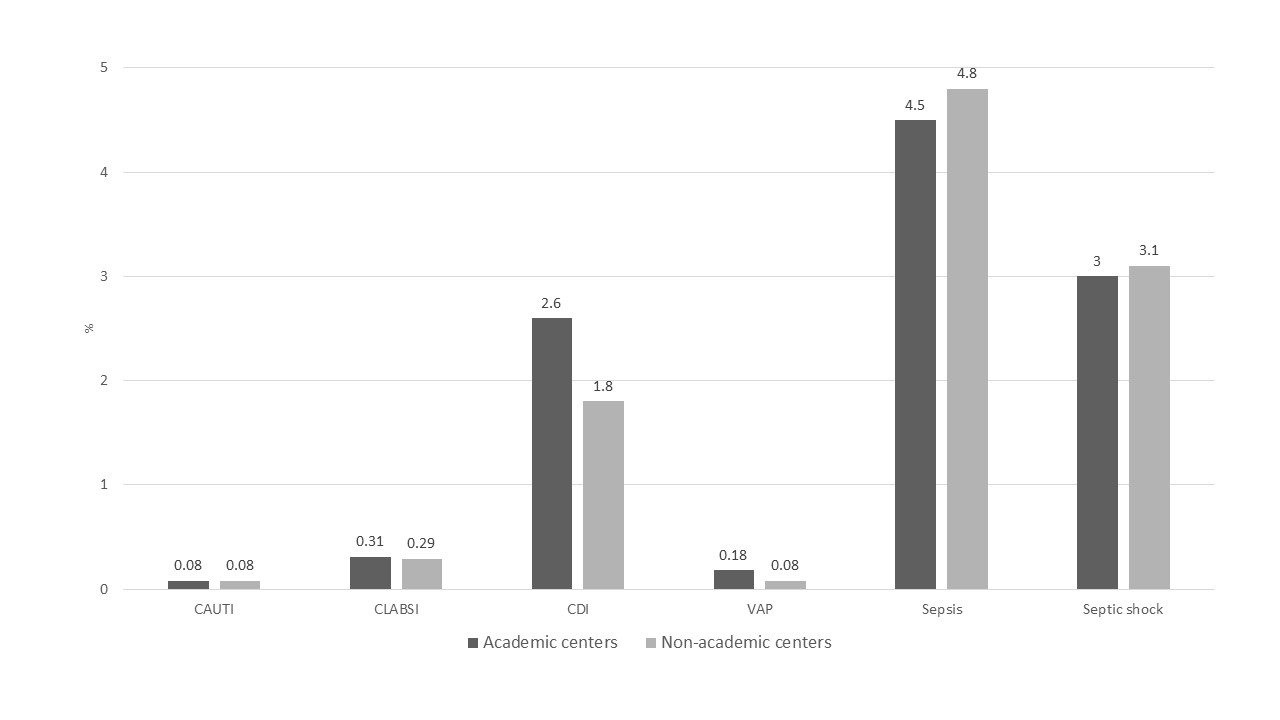

Supplement: Supplementary file 2 [file Image_2.JPEG]

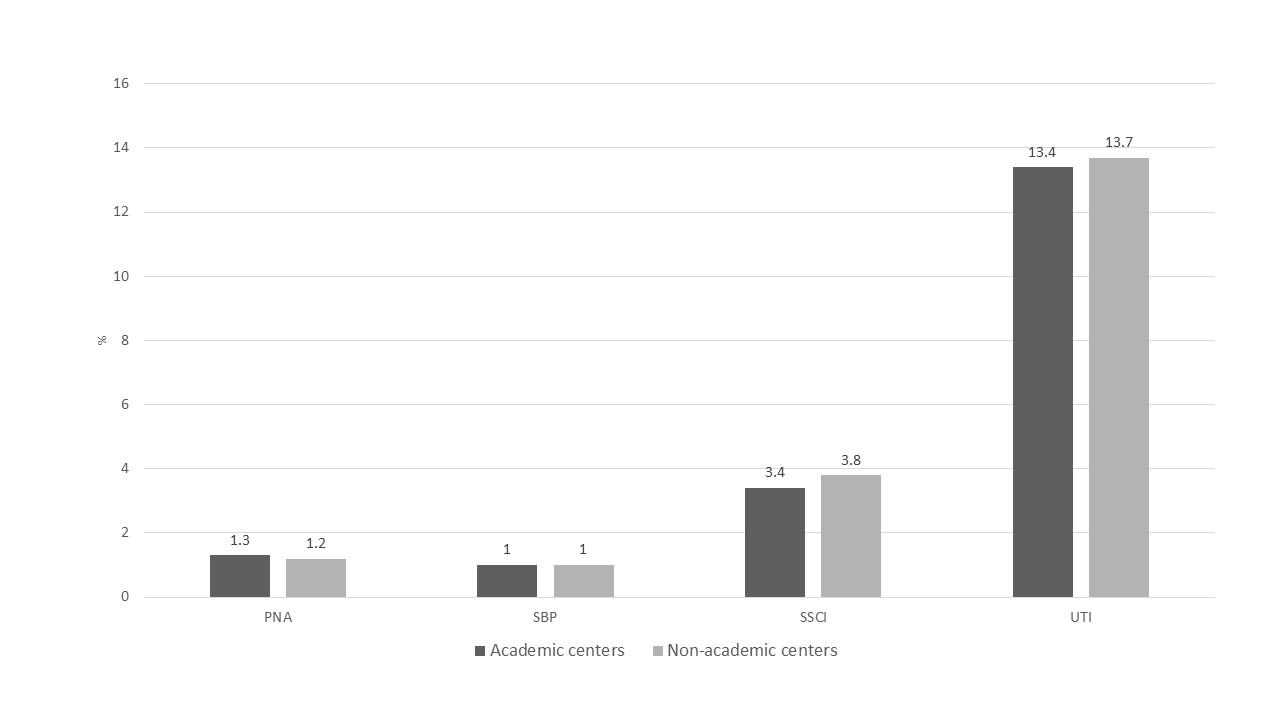

Supplement: Supplementary file 3 [file Image_3.JPEG]

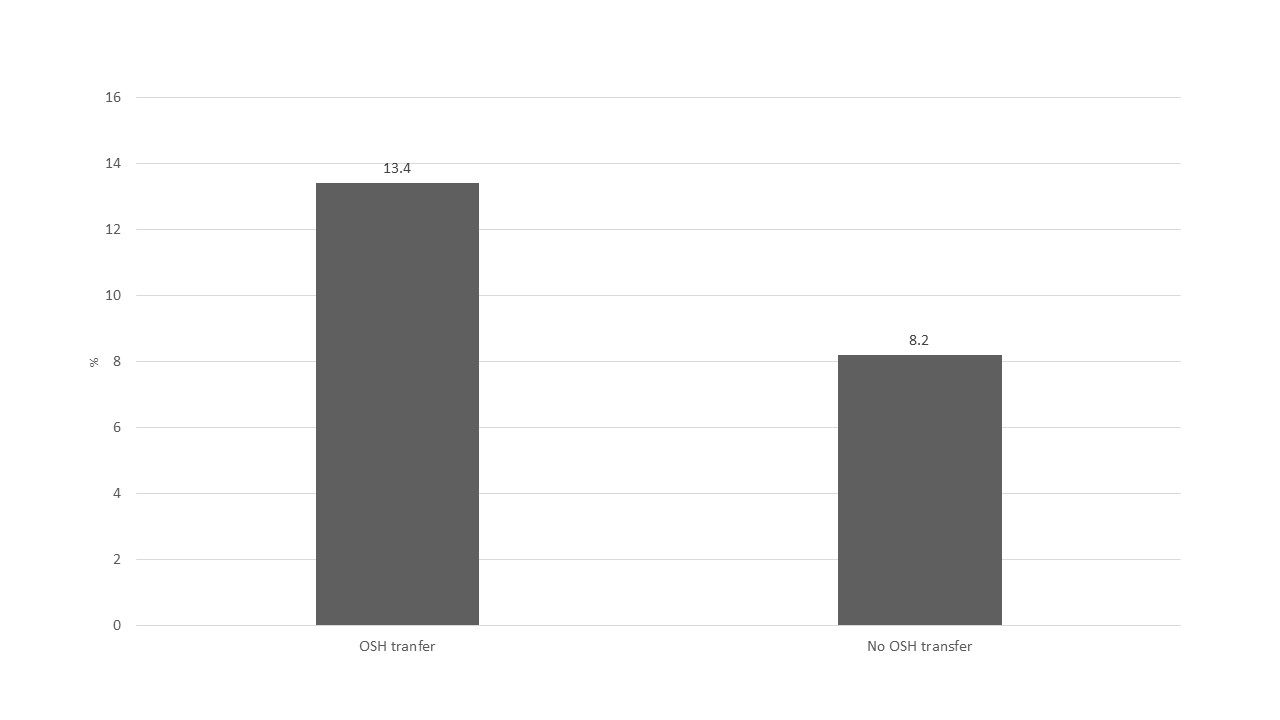

Supplement: Supplementary file 4 [file Image_4.JPEG]

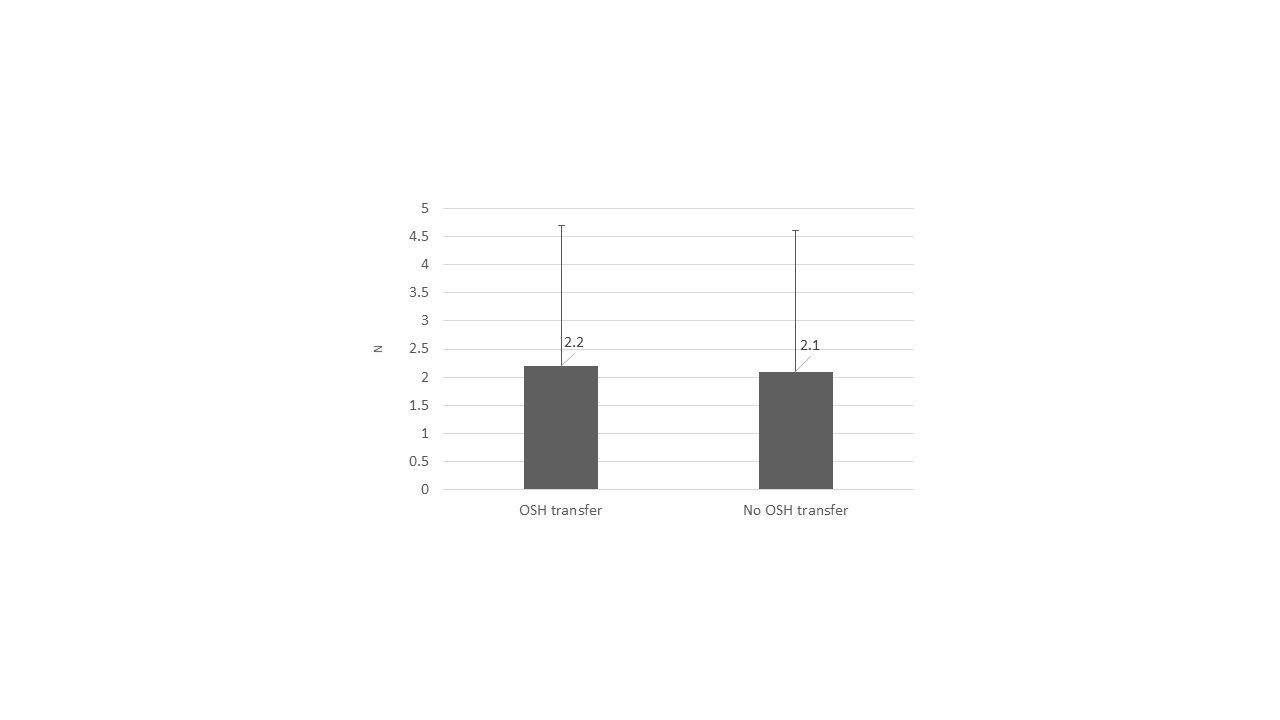

Supplement: Supplementary file 5 [file Image_5.JPEG]
