# Supplementary material for: Higher Frequency of Hospital-Acquired Infections but Similar In-Hospital Mortality Among Admissions With Alcoholic Hepatitis at Academic vs. Non-academic Centers
Source: Front Physiol. 2020 Dec 3;11:594138. doi: 10.3389/fphys.2020.594138 (PMC7744884; doi:10.3389/fphys.2020.594138)
Supplement: Supplementary file 6 [file Table_1.DOCX]

**Supplementary Table 1** ICD-09 codes to stratify admissions with alcoholic hepatitis to organ failure and acute on chronic liver failure (ACLF).

| Diagnosis | ICD-9 |
| --- | --- |
| Cardiovascular | |
| Central venous pressure | 89.62 |
| Pulmonary artery/wedge pressure | 89.64 |
| Arterial line | 89.61 |
| Septic shock | 785.52 |
| Severe sepsis | 995.92 |
| Pulmonary | |
| Mechanical ventilation | 96.7, 96.70, 96.71, 96.72 |
| Renal | |
| Hemodialysis | 39.95 |
| Acute kidney failure | 584.6, 584.7 |
| Brain | |
| Hepatic encephalopathy | 070.0, 070.20, 070.22, 070.21, 070.23, 70.42, 070.43, 070.44, 070.49, 070.6, 070.71 |

**Supplementary Table 2** ICD-09 codes to stratify admissions with alcoholic hepatitis to liver disease complications

| Diagnosis | ICD-09 code |
| --- | --- |
| Variceal bleeding | 456.0, 456.20 |
| Varices | 456.1, 456.21 |
| Ascites | 789.5, 789.59 |
| Hepatic encephalopathy | 572.2 |
| Spontaneous bacterial peritonitis | 567.0, 567.2, 567.21, 567.29, 567.8, 567.89, 567.9 |

| Supplementary Table 3 ICD-09 procedure codes on use of hospital resources   \| Variable \| ICD-9 code \| \| --- \| --- \| \| Palliative care \| V667 \| \| Ventilator \| 96.7, 96.70, 96.71, 96.72 \| \| Hemodialysis \| 39.95 \| \| Blood transfusion \| 99.03, 99.04, 9.05, 99.06 \| \| Endoscopic evaluation \| 42.91, 42.33, 42.24, 43.41 \| \| Liver Transplantation \| 50.51, 50.59 \| \| Central venous pressure monitoring \| 89.62 \| |  |
| --- | --- | --- | --- | --- | --- | --- | --- | --- | --- | --- | --- | --- | --- | --- | --- | --- | --- |

**Legends to Supplementary Figures**

**Supplementary Figure 1** Proportion of patients admitted with alcoholic hepatitis complicated by hospital acquired infections (HAI) or community acquired infections (CAI): comparison of admissions as transfer from outside hospital (OSH) vs. direct admissions to the index hospital.

**Supplementary Figure 2** Proportion of alcoholic hepatitis admissions comparing based on outside hospital (OSH) transfer for **A)** sites of hospital acquired infections: catheter associated UTI (CAUTI), central line associated blood stream infections (CLABSI), C. difficile infection (CDI), ventilator associated pneumonia (VAP), sepsis, and septic shock. **B)** sites of community acquired infections: pneumonia (PNA); spontaneous bacterial peritonitis (SBP); skin and subcutaneous infections (SSCI); and urinary tract infections (UTI).

**Supplementary Figure 3** Comparison of admissions with alcoholic hepatitis based on outside hospital (OSH) transfer for **A)** in-hospital mortality rate and **B)** mean ± SD of number of procedures.
